# Could galcanezumab modulate inflammatory cytokines? A single-centre exploratory study

**DOI:** 10.1007/s00415-026-13706-3

**Published:** 2026-02-24

**Authors:** Giulia Ceccardi, Renata Rao, Francesca Schiano di Cola, Beatrice Fiducia, Chiara Tolassi, Virginia Quaresima, Irene Mattioli, Klaudia Eshja, Elena Cresta, Stefano Gipponi, Andrea Pilotto, Alessandro Padovani

**Affiliations:** 1 Neurology Unit, Ospedale Maggiore di Cremona, ASST-Cremona, 26100 Cremona, Italy; 2 Department of Continuity of Care and Frailty, Neurology Unit, ASST Spedali Civili Hospital, 25123 Brescia, Italy; 3 Department of Clinical and Experimental Sciences, University of Brescia, 25123 Brescia, Italy; 4 Neurobiorepository and Laboratory of Advanced Biological Markers, University of Brescia and ASST Spedali Civili Hospital, 25123 Brescia, Italy; 5 Residency Program in Clinical Pathology and Clinical Biochemistry, Department of Molecular and Translational Medicine, University of Brescia, 25123 Brescia, Italy; 6 Department of Clinical Laboratory, ASST Spedali Civili Hospital, 25123 Brescia, Italy; 7 Department of Neurobiology, Care Sciences and Society (NVS) Center for Alzheimer Research, Division of Clinical Geriatrics, Karolinska Institutet, Stokolhm, Sweden; 8 Brain Health Center, University of Brescia, 25123 Brescia, Italy

**Keywords:** Migraine, Inflammation, Cytokines, Anti-CGRP monoclonal antibodies

## Abstract

**Introduction:**

Migraine is a highly disabling neurological disorder. The pathophysiological mechanisms underlying this condition are complex and not yet fully elucidated. While calcitonin gene-related peptide (CGRP) plays a pivotal role in the genesis of migraine, not all patients respond to CGRP-targeted therapeutic interventions, suggesting the involvement of alternative mechanisms such as neuroinflammation. In this context, cytokines may act as modulators of inflammatory responses associated with migraine. The objective of this study is to evaluate cytokine profiles in patients with episodic and chronic migraine in comparison with healthy controls and to investigate the modulatory effect of galcanezumab treating episodic migraine patients.

**Methods:**

in this prospective pilot study, 17 patients with high-frequency episodic migraine (EM), 18 with chronic migraine (CM), and 17 healthy controls (HC) were enrolled. Plasma cytokine levels (IFN-γ, IL-1β, IL-10, IL-6, TNF-α) were measured using multiplex immunoassay during the interictal phase. Blood samples of the EM group eligible for treatment with galcanezumab were additionally collected at three time points: baseline (T0) and 5 and 27 days after administration of galcanezumab (T5, corresponding to the maximum serum concentration (Cmax), and T27, drug half-life, respectively).

**Results:**

In comparison with patients diagnosed with CM and HC, EM patients demonstrated elevated interictal levels of IFN-γ, IL-1β, IL-6 and IL-10 (*p* < 0.001). In perspective analyses, a significant decrease in cytokine levels was observed in the galcanezumab-treated group at Cmax (T5) for IFN-γ, IL-1β, IL-10, and TNF-α, but not for IL-6 (*p* = *0.640*).

**Discussion:**

This study highlights distinct cytokine profiles associated with chronic and episodic migraine. Treatment with galcanezumab was associated with a transient reduction in both pro- and anti-inflammatory cytokines during the first month of therapy. These findings support the hypothesis that CGRP could influence the inflammatory pathways in migraine pathophysiology.

## Introduction

Headache disorders are the first cause of global disability (expressed as years lived with disability [YLDs]) between young adults, as reported by the Global Burden of disease 2019 study [1], and migraine is the second cause of disability-adjusted life years (DALYs) between neurological diseases [2]. Approximately 15% of the global population is affected, with a particularly high prevalence among young adults [3].

A full understanding of the disease's pathophysiology is essential for the development of optimal treatment strategies and the potential for a cure. The discovery of the CGRP and its role in the pathophysiology of migraine attacks has had a profoundly positive impact on the therapeutic landscape [4]. It is regrettable that the CGRP does not fully elucidate the mechanisms underlying a migraine crisis, as not all patients respond to treatment targeting this molecule. Consequently, alternative potential mechanisms have been investigated. For instance, neuroinflammation may also have a part to play in the regulation of trigeminal pain and the maintenance of pain sensitization. The CGRP molecule has been demonstrated to activate immune cells of innate immunity, including mast cells and dendritic cells, through the trigemino-vascular system, thereby inducing neurogenic inflammation. Conversely, the degranulation of activated mast cells results in the release of significant inflammatory mediators, including histamine, serotonin, and cytokines. These contribute to the sensitization of trigeminal nociceptors [5–7].

Cytokines are important endogenous substances that are involved in immune and inflammatory responses, and as such a significant volume of research has been dedicated to investigating their role during both ictal and interictal states in migraine, with contradictory results [8]. In migraine pathophysiology, a complex interplay between pro- and anti-inflammatory cytokines is observed, contributing to both the initiation and modulation of attacks. Pro-inflammatory cytokines such as tumor necrosis factor-alpha (TNF-α) and interleukin-6 (IL-6) are frequently elevated in the interictal phase, suggesting a persistent low-grade inflammatory state in migraineurs [9]. TNF-α, produced by monocytes, macrophages, and astrocytes, is involved in immune activation and sensitization of the nociceptive pathways [10]. Similarly, IL-6 promotes T- and B-cell activation and has been implicated in trigeminal system sensitization. In contrast, anti-inflammatory cytokines like interleukin-10 (IL-10) and interleukin-4 (IL-4) are generally reduced or unchanged interictally: IL-10 inhibits the production of TNF-α and IL-6, thereby modulating inflammation, while IL-4 plays a role in downregulating pro-inflammatory cytokines and promoting immune homeostasis [11]. The findings indicate an imbalance that is biased toward a pro-inflammatory environment in migraine, which may have a role in the generation and chronification of attacks [8].

Neurogenic inflammation has been proposed to play a role in migraine involving altered cytokine levels, with CGRP being the primary mediator of subtle inflammatory alterations linked to pain [12, 13]. In light of this hypothesis, treatment with anti-CGRP monoclonal antibodies could potentially modulate and reduce inflammatory alterations; however, in vivo analysis of how such therapy affects cytokine levels in a real-world patient cohort has not yet been conducted. The aim of the present study was twofold: (i) to evaluate whether interictal cytokine levels differed across diagnostic groups, namely, patients with episodic migraine (EM) and chronic migraine (CM), and healthy controls (HC); and (ii) to investigate whether treatment of EM with galcanezumab (a monoclonal antibody targeting CGRP ligand) could modulate inflammatory markers longitudinally assessed in plasma.

## Methods

### Study design and participants

This prospective study was conducted at the Headache Centre of the Neurology Clinic at the Spedali Civili Hospital of Brescia and spanned a 6-month period (January–June 2023). The study population comprised adult patients (aged 18–65 years) with a diagnosis of migraine with or without aura, episodic or chronic in frequency, in accordance with the International Classification of Headache Disorders, 3rd edition (ICHD-III)[14]. To be eligible for inclusion, patients were required to be able to sign and comprehend a written informed consent form and to be free from an ongoing migraine crisis at the time of sampling. Patients with a history of pathology involving the immune system or ongoing infection (e.g. upper/lower respiratory tract infection, urinary tract infection) were excluded from participation.

For the primary end point, three groups of patients were selected: patients with CM, HC, and patients with a diagnosis of high-frequency EM (frequency of migraine days/month between 8 and 14 in the preceding three months) eligible for the initiation of galcanezumab.

Data pertaining to the frequency of headaches (expressed as monthly headache days (MHDs)), monthly analgesic medications, migraine disability (measured by the MIDAS score)[15], headache impact test 6 (HIT-6) score [16], and demographic information were collected at the baseline (T0) assessment. The frequency of migraine episodes was recorded via a self-report method utilizing a daily paper headache diary. Blood samples of patients were collected in the interictal phase for the two groups of patients with migraine (CM and EM). For the group of patients in treatment with galcanezumab, blood samples were collected in accordance with the pharmacokinetic characteristics of the drug. The time points for sample collection were as follows:

-T0 (baseline): prior to the first administration of the anti-CGRP monoclonal antibody;
-T5: 5 days after the first administration of galcanezumab, corresponding to the maximum plasma concentration of the drug (Cmax);
-T27: 27 days after the first administration of galcanezumab, corresponding to the half-life of the drug.

Given the preliminary and exploratory nature of this pilot study, a formal power analysis was not performed, and a minimum sample size of 15 patients was defined a priori.

### Study protocol

Following the signature of the written informed consent form, all patients were anonymized, and venous blood samples were collected. A single sample was obtained from CM and HC, whereas in EM samples were collected at baseline (T0), after 5 (T5) and 27 days (T27) after the first administration of galcanezumab. Each sample was centrifuged at 2500* g* for 10 min and subsequently aliquoted into cryovials, each containing 500 μl of plasma/serum, and stored at − 80 °C until the day of analysis. To assess the degree of inflammation, the Human ProcartaPlex Mix&Match 14-plex kit was employed for cytokine levels assessment. The ProcartaPlex assays are multiplex immunoassays that employ the Luminex xMAP (multi-analyte profiling) technology, which enables the simultaneous detection and quantification of up to 80 protein targets in a single 25–50 μL sample. According to the manufacturer’s instructions, on the day of analysis, samples were thawed at room temperature. Subsequently, they were centrifuged at 10,000* g* for 5 min before being dispensed into the plate. All samples within the 14-plex panel were analysed in technical duplicate and all replicates with a coefficient of variation (CV) of less than 20% were included in the analysis. From 14 plex kit, the cytokines most involved in migraine were selected based on literature: IFN ỿ, IL-1β, IL-10, IL-6, and TNFα [8, 17].

Assay sensitivity and dynamic ranges were defined by the manufacturer. Cytokine concentrations below the lower limit of detection were handled according to standard analytical recommendations and were interpreted with caution. Given the exploratory nature of the study and the assessment of small magnitude changes, observed variations in cytokine levels should be considered in light of the intrinsic variability of multiplex immunoassays.

## Statistical analysis

Shapiro–Wilk test was used to ascertain whether variables have a normal distribution. Continuous variables with normal distribution were described as mean and standard deviation otherwise were described as median and interquartile range defined by the 25th and 75th percentiles. Categorical variables were expressed as frequencies and percentages. Statistical differences between groups were evaluated with Kruskal–Wallis test with Dunn’s post hoc comparisons or Mann Whitney *U* test for variable without normal distribution or with Student’s *t* test or ANOVA for variable with normal distribution, as appropriate. Chi-square test was used to compare the frequency of categorical variables between different groups. Outliers were defined by single values outside the upper and lower limits of three standard deviation of the specific group (i.e. CM, EM and HC).

For the secondary end point, Friedman test with Conover’s post hoc test was used to verify the presence of any significant differences in continuous variables in anti-CGRP group at different time points.

Effect size magnitude and uncertainty were addressed separately using *ω*^2^/*ε*^*2*^ and 95% confidence intervals, respectively. Effect sizes for pairwise comparisons were expressed as rank-biserial correlations (*r*_rβ_).

Spearman Rho correlations investigated potential correlations between clinical variables and levels of cytokines.

Given the exploratory nature of the study and the absence of established biological cutoff values for the investigated cytokines, no primary cytokine outcome was pre-specified. Multiple cytokines were therefore analysed in a hypothesis-generating framework. Accordingly, *p*-values are intended to be interpreted descriptively rather than confirmatorily, and no adjustment for multiple comparisons was applied.

Statistical significance was set at *p* < 0.05. Data analyses were carried out with IBPM SPSS Statistics 25.0 per Windows (SPSS Inc., Chicago, IL, USA).

## Results

Fifty-three patients signed the informed consent. As one sample exceeded the upper limits of 3 standard deviations regarding IL-6, it was excluded from the analysis. Then, the sample included 52 subjects, 17 patients with high-frequency EM eligible for galcanezumab, 18 patients with CM, and 17 age-matched HC. Table 1 presents the demographic and clinical characteristics of the three groups under analysis.

**Table 1:** Demographic and clinic characteristic of the three groups: patients with episodic migraine before starting anti-CGRP mAb, patients with chronic migraine, and healthy control subjects

	Patients with EM (*n* = 17)	Patients with CM (*n* = 18)	HC (*n* = 17)	*P* value
Age (years, mean [SD])	43.8 [8.7]	48.1 [13.1]	45.4 [17.5]	0.266*
Sex (female, *n* [%])	14 [82.4]	16 [88.9]	13 [76.5]	0.624^§^
Disease duration (years, mean [SD])	27.5 [8.7]	33.1 [13.1]	Not applicable	0.156*
Monthly headache (days, median [IQR])	10.0 [10.0–12.0]	29.0 [19.3–30.0]	Not applicable	** < *0.001***^†^
Monthly analgesic medication (tablets/months, median [IQR])	12.0 [10.0–15.0]	17.5 [11.5–31.5]	Not applicable	0.108^†^
MIDAS score (median [IQR])	55.0 [46.0–90.0]	103.5 [57.0–146.3]	Not applicable	***0.047***^†^
HIT-6 score (mean [SD])	64.1 [5.1]	64.5 [4.6]	Not applicable	0.804*

*CM* chronic migraine; *EM* episodic migraine; *HC* healthy control; *HIT-6* headache impact test with six-items; *IQR* interquartile range; *MIDAS* migraine disability assessment; *SD* standard deviation.

Categorical variables are expressed as number and percentage, and continuous variables as mean and standard deviation or as median and interquartile range defined by the 25th and 75th percentiles. *P value* < *0.05*.

* ANOVA; ^§^ chi square test; ^†^ Mann Whitney U test

The analysis of plasma cytokines revealed that patients with EM exhibited elevated levels of the examined cytokines, when compared to the chronic migraine group and healthy controls (IFN-ỿ = 0.01 [0.01–2.80] pg/ml vs 0.00 [0.00–0.84] pg/ml vs 0.00 [0.00–0.00] pg/ml *p* = < *0.001*; IL-6 = 0.01 [0.01–0.01] pg/ml vs 0.00 [0.00–0.00] pg/ml vs 0.00 [0.00–0.00] pg/ml *p* = < *0.001*; IL-1β = 0.01 [0.01–0.98] pg/ml vs 0.00 [0.00–0-00] pg/ml vs 0.00 [0.00–0.45] pg/ml *p* = < *0.001*; IL-10 = 0.30 [0.12–0.39] pg/ml vs 0.05 [0.00–0.08] pg/ml vs 0.06 [0.00–0.14] pg/ml *p* = < *0.001*). Levels of TNF-α did not reach statistical significance (TNF-α = 0.29 [0.01–1.53] pg/ml vs 0.23 [0.00–0.28] pg/ml vs 0.00 [0.00–0.38] pg/ml *p* = *0.057*) (See Table 2).

**Table 2:** Comparison of the plasma cytokines levels between the three groups (patients with episodic migraine before starting anti-CGRP mAb, patients with chronic migraine, and healthy control subjects)

	Patients with EM (*n* = 17)	Patients with CM (*n* = 18)	HC (*n* = 17)	*P value* *(ε*^*2*^*)*	*P value* EM and CM (*r*_rβ_)	*P value* EM and HC (*r*_rβ_)	*P value* CM and HC (*r*_rβ_)
*IFN-ỿ, *pg/ml median, ([25th–75th percentiles], [CI 95%])	0.01([0.01–2.80], [1.20–5.41])	0.00 ([0.00–0.84], [0.10–3.02])	0.00 ([0.00–0.00], [0.00–2.69])	** < *0.001*** *(0.379)*	** < *0.001*** *(0.813)*	***0.002*** (0.536)	*0.221* *(0.196)*
*IL-6, *pg/ml median, ([25th–75th percentiles], [CI 95%])	0.01 ([0.01–0.01], [0.00–3.32])	0.00 ([0.00–0.00], [0.00–11.73])	0.00 ([0.00–0.00], [0.00–13.94])	** < *0.001*** *(0.453)*	** < *0.001*** *(0.686)*	** < *0.001*** *(0.779)*	*0.727* *(0.046)*
*TNF-α, *pg/ml median, ([25th–75th percentiles], [CI 95%])	0.29 ([0.01–1.53], [0.31–1.49])	0.23 ([0.00–0.28], [0.02–0.35])	0.00 ([0.00–0.38], [0.02–1.04])	*0.057* *(0.112)*	*0.064* *(0.356)*	***0.025*** *(0.443)*	*0.667* *(0.082)*
*IL-1β, *pg/ml median, ([25th–75th percentiles], [CI 95%])	0.01([0.01–0.98], [0.11–1.10])	0.00 ([0.00–0–00], [0.00–0.32])	0.00 ([0.00–0.45], [0.05–0.76])	** < *0.001*** *(0.292)*	** < *0.001*** *(0.765)*	***0.045*** *(0.329)*	*0.067* *(0.294)*
*IL-10*, pg/ml median, ([25th–75th percentiles], [CI 95%])	0.30 ([0.12–0.39], [0.02–0.58]	0.05 ([0.00–0.08], [0.00–0.01])	0.06 ([0.00–0.14], [0.00–0.02])	** < *0.001*** *(0.348)*	** < *0.001*** *(0.788)*	***0.003*** *(0.599)*	*0.275* *(0.225)*

*CM* Chronic migraine; *EM* episodic migraine; *HC* healthy control; *IQR* interquartile range defined by the 25th and 75th percentiles; *CI* confidence interval. *P value* < *0.05 *(Kruskal–Wallis test)

**Fig. 1:**
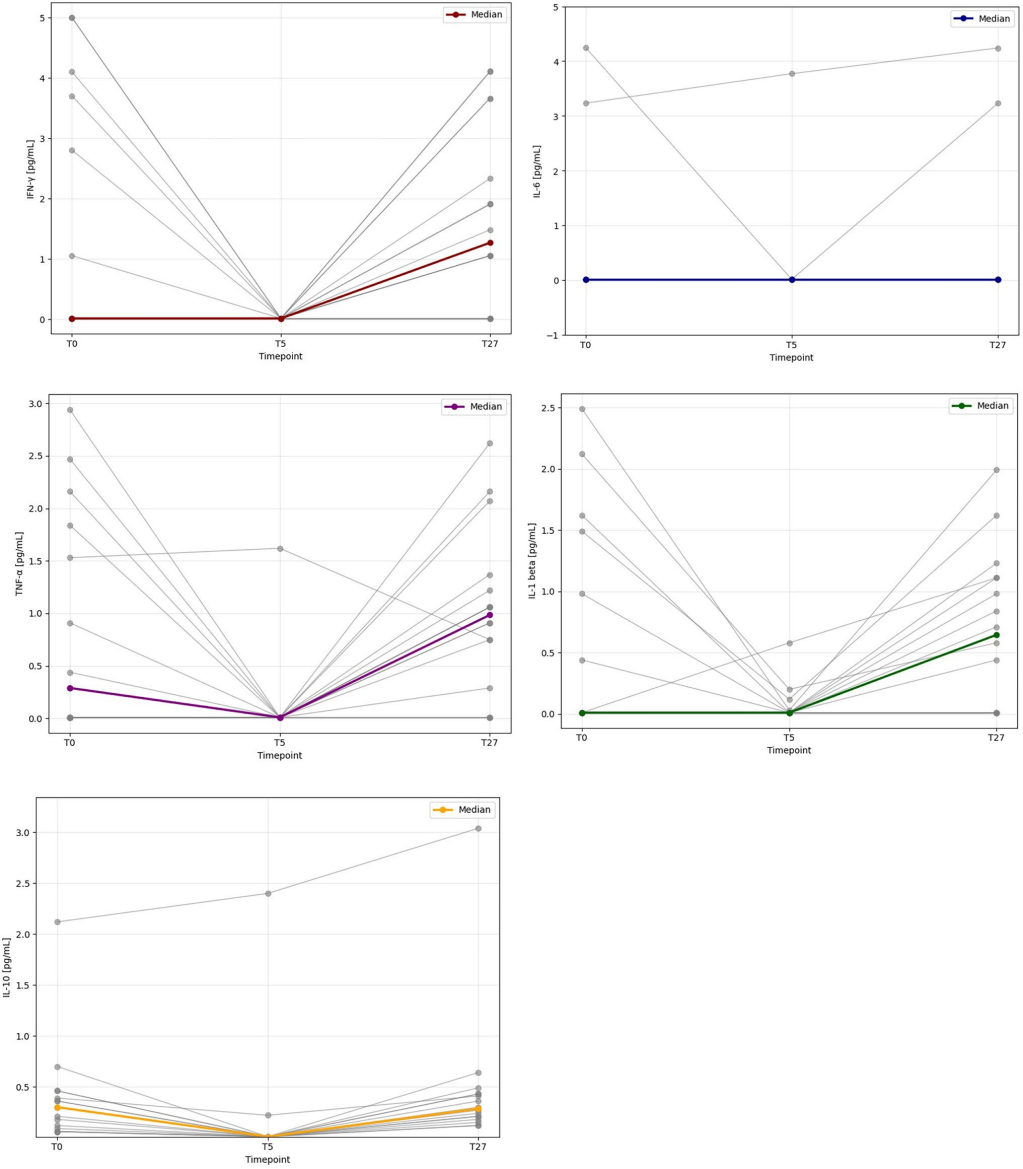
Cytokines plasma levels (pg/ml) at baseline (T0), Cmax (T5), and half-live (T27) of treatment with galcanezumab

Regarding the secondary outcome, the analysis was conducted on 16 subjects due to the lack of a blood sample of 1 subject at T5. The plasma concentrations of the analysed cytokines exhibited an inverse correlation with the pharmacokinetic profile of galcanezumab, displaying lower levels at the Cmax if compared to baseline and half-life (Fig. 1).

The Friedman test showed a statistically significant difference in all cytokines except for IL-6 at Cmax compared to the value at baseline and half-life (IFN-ỿ *p* = *0.002*; IL-6 *p* = *0.640*; TNF-α *p* = *0.001*; IL-1β *p* = *0.009*; IL-10 *p* < *0.001*) (see Table 3).

**Table 3:** Comparison between cytokines levels at baseline (T0), Cmax (T5), and half-live (T27)

	Baseline (T0)	Cmax (T5)	Half-life (T27)	*p value (*ω^2^)	*p-value* T0 – Cmax (r_rβ_)	*p-value* Cmax– half-life (r_rβ_)	*p-value* T0– half-life (r_rβ_)
*IFN-ỿ, pg/ml* median, ([25th–75th percentiles], [CI 95%])	0.01 ([0.01–3.05], [1.45–5.55])	0.01 ([0.01–0.01], [0.00–0.00])	1.27 ([0.01–2.66], [1.13–3.18])	***0.002*** *(0.173)*	***0.014*** *(1.000)*	** < *0.001*** *(− 1.000)*	*0.084* *(− 0.359)*
*IL-6, pg/ml* median, ([25th–75th percentiles], [CI 95%])	0.01 ([0.01–0.01], [0.00–3.23])	0.01 ([0.01–0.01], [0.00–2.30])	0.01 ([0.01–0.01], [0.00–3.44])	*0.640* *(0.000)*	*0.826* *(0.333)*	*0.277* *(− 1.000)*	*0.383* *(0.000)*
*TNF-α, pg/ml* median, ([25th–75th percentiles], [CI 95%])	0.29 ([0.01–1.61], [0.37–1.55])	0.01 ([0.01–0.01], [0.00–0.42])	0.99 ([0.99–1.26], [0.225–0.914])	***0.001*** *(0.180)*	***0.026*** *(0.956)*	** < *0.001*** *(− 0.934)*	*0.058* *(− 0.410)*
*IL-1β, pg/ml* median, ([25th–75th percentiles], [CI 95%])	0.01 ([0.01–1.11], [0.22–1.15])	0.01 ([0.01–0.02], [0.00–0.05])	0.65 ([0.01–1.11], [0.19–0.59])	***0.009*** *(0.127)*	***0.046*** *(0.857)*	** < *0.001*** *(− 1.000)*	*0.102* *(− 0.192)*
*IL-10, pg/ml* median, ([25th–75th percentiles], [CI 95%])	0.30 ([0.11–0.41], [0.02–0.59])	0.01 ([0.01–0.01], [0.00–0.93])	0.29 ([0.20–0.43], [0.01–1.26])	** < *0.001*** *(0.040)*	***0.001*** *(0.868)*	** < *0.001*** *(− 1.000)*	***0.005*** *(− 0.500)*

*CI* confidence intervals. Continuous variables are expressed as median and interquartile range defined by the 25th and 75th percentiles. *P value* < *0.05* using Friedman test with Conover’s post hoc test. Analysis done on 16 patients (1 missing datum at T5)

After 3 months of treatments, 10/14 patients responded to anti-CGRP therapy (≥ 50% reduction in monthly headache days at 3 months compared to baseline). No statistically significant differences emerged investigating the correlation between clinical response and levels of cytokines at baseline (see Table 4).

**Table 4:** Correlations between levels of cytokines at baseline (T0) and clinical response to treatment with galcanezumab after 3 months

	Responders to treatment after 3 months of galcanezumab (10/14 pts)	*p*-value
*IFN-ỿ T0*		
Spearman Rho	*− *0.295	*0.306*
*IL-6 T0*		
Spearman Rho	*− *0.225	*0.439*
*TNF-α T0*		
Spearman Rho	*− *0.111	*0.694*
*IL-1β T0*		
Spearman Rho	*− *0.025	*0.934*
*IL-10 T0*		
Spearman Rho	*− *0.217	*0.456*

Responder patients are those who reach a reduction > = 50% of monthly headache days after 3 months of treatment compared to baseline. *P value* < *0.05*

## Discussion

The study revealed distinct inflammatory profiles in patients with migraine, with a focus on the different clinical conditions (EM, CM, HC). The study also showed patients’ cytokine profile during the first month of treatment with galcanezumab.

Our study highlighted significantly higher IL-10 levels in patients with episodic migraine (EM) compared to those with chronic migraine (CM) and healthy controls (HC). A recent systematic review [8] examined pro- and anti-inflammatory cytokines in migraine during both ictal and interictal phases. With respect to IL-10, most studies reported lower or comparable interictal levels in migraineurs relative to controls, while a few observed increased levels during attacks [17–19].

Given these inconsistencies, no definitive conclusions can be drawn. With our findings, however, we could hypothesize a potential upregulation of IL-10 in EM, followed by downregulation or diminished activity in CM, where central sensitization predominates.

Regarding pro-inflammatory cytokines, TNF-α, IFN-γ, IL-6, and IL-1β levels were higher in EM patients compared to HC, whereas CM patients did not show significant differences compared with HC. The EM group showed IL-6 level elevation in comparison to both the HC and CM groups, despite the fact that the absolute median values were found to be low in all three groups. This finding is at odds with those of previous studies, which reported higher levels in CM than in EM [20]. In the present cohort, the observed divergence may be attributable to the limited sample size. Further studies employing a larger sample are required to substantiate the result.

Moreover, in our sample TNF-α had higher absolute median levels in EM and CM versus HC (*p value* is statistically significant only for EM vs HC). This cytokine is produced by the degranulation of mast cells and by other innate immune cells (including dendritic cells and macrophages) activated by CGRP [7]. This mechanism may help to contextualize the elevated TNF-α levels observed in EM and CM within our sample. However, in the absence of established cutoff values in the existing literature, definitive conclusions cannot be drawn, as the observed findings may reflect modest variations within physiological ranges.

The present exploratory findings contribute to the growing and heterogeneous recent literature that seeks to deepen and investigate the pathophysiology of migraine. However, further research is required to ascertain the precise role of cytokines. Rigorous, reproducible, homogeneous, and large-scale studies are essential for developing robust results and establishing cutoff values for the use of cytokines as biomarkers for migraine.

Focusing on the secondary outcome, to the best of our knowledge, this study is one of the first to investigate a potential temporal correlation between interictal plasma cytokine levels and anti-CGRP treatment. All cytokines analysed in our cohort, including both pro- and anti-inflammatory molecules, showed a reduction in concentration in accordance with the elevated plasma concentration of galcanezumab. In particular, TNF-α and IL-10 showed a noticeable decrease at T5 and went back to baseline value at T27 while IL-6 remained stable in all three time points.

Ray and colleagues were the first to demonstrate that in CM patients, after three months of treatment with anti CGRP (fremanezumab and galcanezumab), no significant change in cytokine expression emerged. In their study, a significant reduction in IL-5 levels was shown in a post hoc analysis, but the interpretation of this funding needs furthers studies [21].

However, literature data clearly shows that CGRP plays many roles in the immune response: CGRP-containing C-nerve fibres are closely related to many immune cells (dendritic cells, mast cells and T cells). CGRP therefore acts as a mediator of neuroimmune communication. Its link with individual immune cells and their interplay remains complex to clarify, but its direct or indirect role in neuroinflammation seems undeniable [7, 22, 23].

The findings of this study suggest that CGRP exerts a regulatory effect on pro- and anti-inflammatory cytokines. However, the restricted sample size and the absence of a control group preclude the drawing of definitive conclusions. It remains uncertain whether the observed downregulation is merely a temporal association or a genuine CGRP-mediated modulation of inflammation. Given the numerous actors involved in migraine neuroinflammation, it seems to be simplistic to claim that CGRP could be a master immunomodulator able to downregulate both pro- and anti-inflammatory cytokines. Moreover, in the absence of compelling evidence to the contrary, it is reasonable to hypothesize that CGRP exerts an influence on IL-6.

Nevertheless, the potential implications of such interesting findings are evident but definitively need further larger studies exploring the direct/indirect impact of inflammatory modulation on symptoms and on multimodal markers, especially of glial activations, which have been primarily evaluated in CM with contrasting results [24].

The fast advancement in automatic detection of multimodal markers of central nervous system definitively needs to be implemented also in migraine research, as it will allow a deeper understanding of biomarkers’ value at single-subject level [25].

This study has several limitations that should be carefully considered. First, the sample size was relatively small, which limits statistical power and the generalizability of the findings.

To promote cohort homogeneity and minimize variability, all participants were treated with galcanezumab, rather than including different anti-CGRP monoclonal antibodies. Consequently, it remains uncertain whether the observed inflammatory patterns are applicable to other agents targeting the CGRP pathway. In addition, galcanezumab is uniquely administered with an initial loading dose; therefore, the present findings may reflect the pharmacokinetic characteristics specific to this agent, and it cannot be excluded that this aspect contributed to the observed results.

Moreover, several clinical factors with potential relevance to inflammatory markers were not accounted for in the analysis. These include the use of concomitant medications (such as preventive therapies), the quantity of nonsteroidal anti-inflammatory drug (NSAID) consumption, comorbidities (obesity, depression), and the menstrual cycle phase in female participants. The absence of these data may have influenced cytokine measurements.

Finally, the follow-up period was limited to the first month of treatment, which constrains the interpretation of longer-term effects, including sustained clinical response, tolerability, and potential persistence of cytokine modulation.

## Conclusion

This exploratory study provides preliminary evidence that patients with EM could exhibit a distinct pro- and anti-inflammatory cytokine profile compared to those with CM and HC, with notably elevated interictal levels of IL-10, IFN-γ, IL-6, and IL-1β. Furthermore, treatment with galcanezumab, a monoclonal antibody targeting the CGRP ligand, seemed associated with a transient reduction in both pro- and anti-inflammatory cytokines during the early phase of therapy. These findings suggest that CGRP may influence inflammatory pathways in migraine pathophysiology beyond its well-known role in nociceptive signalling.

To ensure cohort homogeneity and reduce treatment-related variability, galcanezumab was intentionally selected among available anti-CGRP monoclonal antibodies. While this approach strengthens internal validity, it limits the generalizability of the results to other agents in the same class. Additional limitations include the small sample size, the lack of a control group, and short follow-up duration. Future studies with larger cohorts, longer observation periods (at least 3–6 months of treatment), comparative designs across different monoclonal antibodies, and matched control group are needed to clarify the role of CGRP-targeted treatments in modulating immune responses in migraine.

## Data Availability

The data and materials are available from the corresponding author upon reasonable request.

## Declarations

### clinical IMPLICLINICAL implication

- Galcanezumab could elicit a modulation of inflammatory cytokines.
- Anti-inflammatory and pro-inflammatory cytokines have a dynamic course and follow the migraine phase (episodic vs chronic).
